# Supplementary material for: Tracking transcription–translation coupling in real time
Source: Nature. 2024 Dec 4;637(8045):487–95. doi: 10.1038/s41586-024-08308-w (PMC11711091; doi:10.1038/s41586-024-08308-w)
Supplement: Supplementary file 1 — Supplementary figure with all uncropped gels; Supplementary Tables 1–3, listing DNA and RNA constructs and oligonucleotides. [file 41586_2024_8308_MOESM1_ESM.pdf]

nature portfolio

https://doi.org/10.1038/s41586-024-08308-w

Supplementary information

Tracking transcription–translation coupling in real time

In the format provided by the authors and unedited

SUPPLEMENTARY INFORMATION

Tracking transcription-translation coupling in real-time

Nusrat Shahin Qureshi[1], Olivier Duss[1*]

Structural and Computational Biology Unit, European Molecular Biology Laboratory, 69117 Heidelberg, Germany

Content:

Supplementary Figure 1: Uncropped gel images

Supplementary Table 1: DNA sequences

Supplementary Table 2: mRNA sequences

Supplementary Table 3: Sequences of DNA oligonucleotides

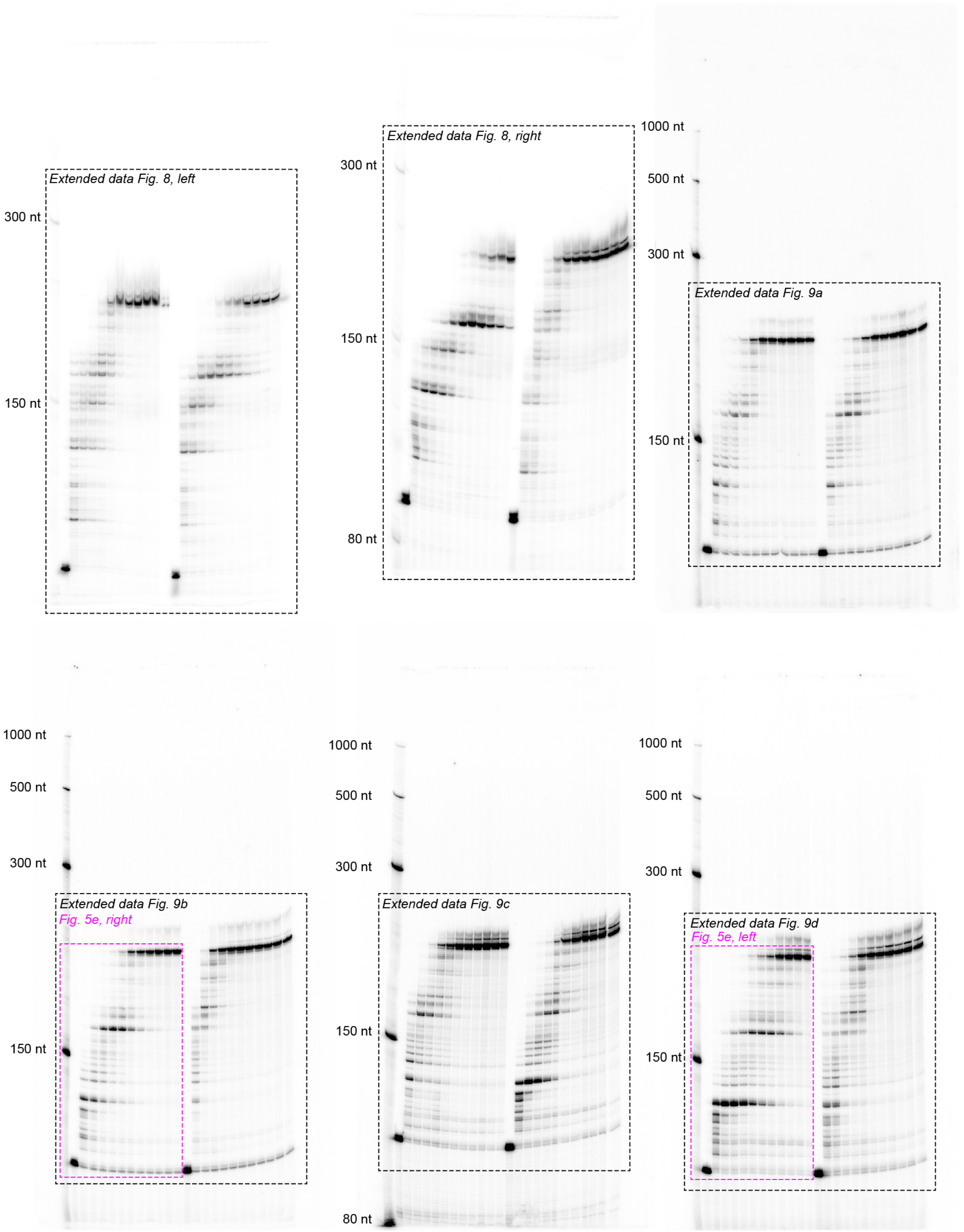

Supplementary Figure 1 | Uncropped transcription assay gel images. Cropped regions are indicated with dashed boxes.

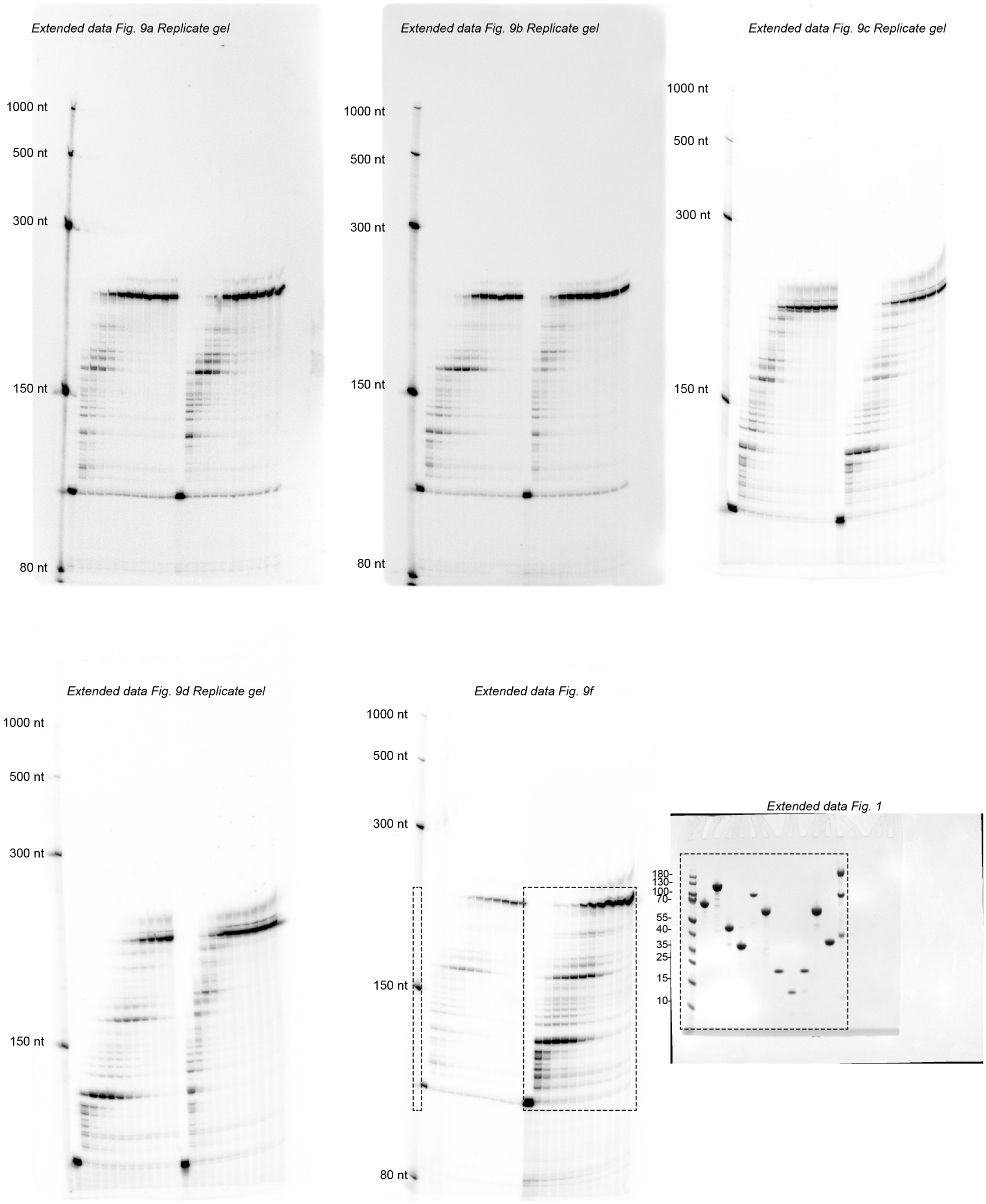

Supplementary Figure 1 (continued) | Uncropped transcription assay gel images and SDS-PAGE gel of all protein factors. Cropped regions are indicated with dashed boxes.

Supplementary Table 1: Overview of artificial DNA constructs used in this study

All used DNA sequences have the following common backbone:

RNAP P1 promotor – 5’-immobilization sequence – ribosome binding site – sequence of interest (see below table) – potential binding site for labeled DNA oligo binding to 3’-end of nascent mRNA – T7 terminator sequence

5’-CTGGCAGTTTTAGGCTGATTTGGTTGAATGTTGCGCGGTCAGAAAATTATTTTA AATTTCCTCTTGTCAGGCCGGAATAACTCCCTATAATGCGCCACC – ACTAAAAGA AGAAGAAAGAGAAA – TAGAAGTAATTTTGTTTAAATTTAAGAAGGAGATAT AAA T – SEQUENCE OF INTEREST (flanked by AUG start codon and TAA stop codon) – CCCTATCCCTTATCTTAAC (F1) **OR** AACCACUCCAAUUACAUACACC (F2) – TAATCACACTGGCTCACCTTCGGGTGGGCCTTTCTGCGTTTAT-3’

DNA construct	Sequence of interest (5’-3’)
prNQ091	ATG GTT ATT ATT GTT GTT ATT ATT GTT ATT ATT GAT TTG GTG AGA GGC ATC AGT ACC GTT ACC ATC GTG ACT ATC ACC GTT ACC ACT ATC GTT TAA
prNQ215	ATG TGT GAA AAG AAT GAT TTG GTG GAA GCC CAA AAT AAA TTT GTG AAT ATT CTG TTC GAG ATC CTG GCA CGT TGG AGT TAT GAG TTT CAT CGT CAA TAA
prNQ216	ATG ATT AAA AAT GTG GAA GAT AAT TAT GTT AAA GAT TTG GTG GAA GCC CTG AAT TTT TGT GAG ATT TGG CAT CCG AGC ACC GTG ATG CAG TTT CAA TAA
prNQ219	ATG AAG AAA GAA GTG AAA GTG ATT AAA GAA AAA GTG AAG ATT AAG GAA AAA AAT GTG AAA TTT GAA GTG AAA GAT TTG GTG GAA GCC CAG GTG ACC CAG AAA GAT TTT CTG TGC TTT CAC AGC CGT GTG AAA ATT CTG TAA
prNQ291	ATG TGT GAA AAG AAT AGT GTG AAG GAA GAG GTT AAT GAA ATT AAA ATT ATT AAA AAA ATT ATT GTT ATT ATT GAT AAT TAT GTT AAA AAG AAA GAA GTG AAG GAT GAA GTG TTT AAG AAA GAA GTG AAA GTG ATT AAA GAA AAA GTG AAG ATT AAG GAA AAA AAT GTG AAA GAA GTG AAA GAT TTG GTG GAA GCC CAA AAT AAA TTT GTG AAT ATT CTG TTC GAG ATC CTG GCA CGT TGG AGT TAT GAG TTT CAT CGT CAA TAA
prNQ301	ATG TGT GAA AAG AAT GAA TTA TAT AAT AAG GTT ATT GAA ATG TAT ATG TAT GAT AAA GAA AGT GGT TTA GAA AAA TGG GTG GTT TTG TGG ATT TTA AAA AAG GAA AAG ATG ATT AAA AGT TGT GGT GTT ATG GAA ATT TTA TTA GTG TTA GAT GTA ATT GAA AAT GTA ATT AAT AAA GAG AAG ATG GAG TTA TGG TGT GTT TGG GGG AGT AAT AAA AAG GAT GAA GTG TTT AAG AAA GAA GTG ATG AAA AAT TAT TAT AAA AAG GGA GGA AAA GTA AAT GAA AAA TAT GAT ATT GTG TTA AAT GAA AAA GTT GTA TTA AAA GTG ATT GAG AAA TTG TAT AAG TGG AAA GGT GAA AGT GTA ATT GTG GGG TAT AAA GAT AAG AAA GAA GAA AAT TTG GGT GAA AAG AGT GGA GAT TTA GTT TTA ATG ATT TTG AGT ATT GGT GTG GAT TTG GTG GAA GCC CAA AAT AAA TTT GTG AAT ATT CTG TTC GAG ATC CTG GCA CGT TGG AGT TAT GAG TTT CAT CGT CAA TAA

Supplementary Table 2: Overview of transcribed mRNAs

Below table shows transcribed mRNA sequences. To calculate the intervening mRNA sequence we counted the nucleotides from the ribosome P-site codon till the active site of the RNAP, as defined by Weixlbaumer. Stalling nucleotide is underlined. Note: Zenkin and co-workers counted from A-site codon till the active site (- 3 nt when comparing to us) and Ebright and co-workers counted the codons from A-site till RNA exit channel (- 17 nt when comparing to us).

These are the transcribed RNA sequences:

DNA construct	Transcribed mRNA sequence (5'-3')	Relevant mRNA lengths
prNQ215<br>**mRNA-28**<br>(F1)	ACU AAA AGA AGA AGA AAG AGA AAU AGA AGU AAU UUU GUU UAA AUU<br>UAA GAA GGA GAU AUA AAU AUG UGU GAA AAG AAU GAU UUG GUG GAA<br>GCC CAA AAU AAA UUU GUG AAU AUU CUG UUC GAG AUC CUG GCA CGU<br>UGG AGU UAU GAG UUU CAU CGU CAA UAA CCC UAU CCC UUA UCU UAA<br>CUA AUC ACA CUG GCU CAC CUU CGG GUG GGC CUU UCU GCG	-91 nt (stalled TEC)<br>-219 nt (Full-length)<br>-**28** nt between P-site and active site at start<br>-156 nt between P-site and active site at TC end
prNQ216<br>**mRNA-46**<br>(F1)	ACU AAA AGA AGA AGA AAG AGA AAU AGA AGU AAU UUU GUU UAA AUU<br>UAA GAA GGA GAU AUA AAU AUG AUU AAA AAU GUG GAA GAU AAU UAU<br>GUU AAA GAU UUG GUG GAA GCC CUG AAU UUU UGU GAG AUU UGG CAU<br>CCG AGC ACC GUG AUG CAG UUU CAA UAA CCC UAU CCC UUA UCU UAA<br>CUA AUC ACA CUG GCU CAC CUU CGG GUG GGC CUU UCU GCG	-109 nt (stalled TEC)<br>-219 nt (Full-length)<br>-**46** nt between P-site and active site at start<br>-71 nt intervening to P1<br>-106 nt intervening to P2<br>-156 nt between P-site and active site at TC end
prNQ219<br>**mRNA-85**<br>(F1)	ACU AAA AGA AGA AGA AAG AGA AAU AGA AGU AAU UUU GUU UAA<br>AUU UAA GAA GGA GAU AUA AAU AUG AAG AAA GAA GUG AAA GUG AUU<br>AAA GAA AAA GUG AAG AUU AAG GAA AAA AAU GUG AAA UUU GAA GUG<br>AAA GAU UUG GUG GAA GCC CAG GUG ACC CAG AAA GAU UUU CUG UGC<br>UUU CAC AGC CGU GUG AAA AUU CUG UAA CCC UAU CCC UUA UCU UAA<br>CUA AUC ACA CUG GCU CAC CUU CGG GUG GGC CUU UCU GCG	-148 nt (stalled TEC)<br>-261 nt (Full-length)<br>-**85** nt between P-site and active site at TC start<br>-198 nt between P-site and active site at TC end
prNQ291<br>**mRNA-193**<br>(F2)	ACU AAA AGA AGA AGA AAG AGA AAU AGA AGU AAU UUU GUU UAA AUU<br>UAA GAA GGA GAU AUA AAU AUG UGU GAA AAG AAU AGU GUG AAG GAA<br>GAG GUU AAU GAA AUU AAA AUU AUU AAA AAA AUU AUU GUU AUU AUU<br>GAU AAU UAU GUU AAA AAG AAA GAA GUG AAG GAU GAA GUG UUU AAG<br>AAA GAA GUG AAA GUG AUU AAA GAA AAA GUG AAG AUU AAG GAA AAA<br>AAU GUG AAA GAA GUG AAA GAU UUG GUG GAA GCC CAA AAU AAA UUU<br>GUG AAU AUU CUG UUC GAG AUC CUG GCA CGU UGG AGU UAU GAG UUU<br>CAU CGU CAA UAA AAC CAC UCC AAU UAC AUA CAC CUA AUC ACA CUG<br>GCU CAC CUU CGG GUG GGC CUU UCU GCG	-256 nt (stalled TEC)<br>-387 nt (Full-length)<br>-**193** nt between P-site and active site at start<br>-324 nt between P-site and active site at end
prNQ301<br>**mRNA-457**<br>(F2)	ACU AAA AGA AGA AGA AAG AGA AAU AGA AGU AAU UUU GUU UAA AUU<br>UAA GAA GGA GAU AUA AAU AUG UGU GAA AAG AAU GAA UUA UAU AAU<br>AAG GUU AUU GAA AUG UAU AUG UAU GAU AAA GAA AGU GGU UUA GAA<br>AAA UGG GUG GUU UUG UGG AUU UUA AAA AAG GAA AAG AUG AUU AAA<br>AGU UGU GGU GUU AUG GAA AUU UUA UUA GUG UUA GAU GUA AUU GAA<br>AAU GUA AUU AAU AAA GAG AAG AUG GAG UUA UGG UGU GUU UGG GGG<br>AGU AAU AAA AAG GAU GAA GUG UUU AAG AAA GAA GUG AUG AAA AAU<br>UAU UAU AAA AAG GGA GGA AAA GUA AAU GAA AAA UAU GAU AUU GUG<br>UUA AAU GAA AAA GUU GUA UUA AAA GUG AUU GAG AAA UUG UAU AAG<br>UGG AAA GGU GAA AGU GUA AUU GUG GGG UAU AAA GAU AAG AAA GAA<br>GAA AAU UUG GGU GAA AAG AGU GGA GAU UUA GUU UUA AUG AUU UUG<br>AGU AUU GGU GUG GAU UUG GUG GAA GCC CAA AAU AAA UUU GUG AAU<br>AUU CUG UUC GAG AUC CUG GCA CGU UGG AGU UAU GAG UUU CAU CGU<br>CAA UAA AAC CAC UCC AAU UAC AUA CAC CUA AUC ACA CUG GCU CAC<br>CUU CGG GUG GGC CUU UCU GCG	-520 nt (stalled TEC)<br>-651 nt (Full-length)<br>-**457** nt between P-site and active site at start<br>-588 nt between P-site and active site at end

Supplementary Table 3: Overview of DNA and RNA oligonucleotides used for biochemical and/or single-molecule assays.

All DNA oligonucleotides were purchased from IDT or Biomers.

Oligonucleotide ID	DNA/RNA sequence (5’-3’)
p0030_ab_fw	TCACGAAAGCTGAGTAGTCACGAGTCTTCT/idSp/CTGGCAGTTTTAGGCTGATTTGG
p0075_ab_bw	CCTTAATCATACTCACCAAATTACCATCCC/idSp/ATAAACGCAGAAAGGCCCAC
p0088_2xCy3.5	Cy3p5-GGGATGGTAATTTGG[dT-Cy3p5]GAGTATGATTAAGG
p0109-biotin	5BiotinTEG/TTATCCGCTCACAATTCCACA
prNQ087-Cy3	GGGAGATCAGGATA/3Cy3Sp/
prNQ088-Cy5	GAGGCCGAGAAGTG/3Cy5Sp/
prNQ127	TTTCTCTTTCTTCTTCTTTTAGTTGTGGAATTGTGAGCGGATAA
prNQ159-Cy3B	GGGAGATCAGGATA-CY3B
prNQ302	GAGGCCGAGAAGTGAAAAACCACTAGTCCACCGCAGCCC
prNQ303	TACCTATGGATCCATATCTGGGGCTGCGGTGGACTAGTGG
prNQ304	CAGATATGGATCCATAGGTATGTGGAATTGTGAGCGGATAA
prNQ331	TTAACATAATTATCT
prNQ341	CACTTCAAATTTCACATTTTTTTCCTTAATCTTCACTTTTTCTTTAATCAC
prNQ345	CATAATTATCAATAATAACAATAATTTTTTTAATAATTTTAAAAAAATCTTCACTTTTTCTTT AATCACTTTCAC
prNQ347	TTCTAAACCACTTTCTTTATCATACATATAAAAAATAAATCTCCACTCTTTTCACCCAAATTT TC
6(FK) mRNA	GGACUACCACCACCCAACCAACACACCCCCGGUAAGGAAAUAAAA**AUG**UUCAAAUUCAAAUUC AAAUUCAAAUUCAAAUUCAAAUAAUUUUUUUUUUUU

/idSp/ denotes abasic site.
[dT-Cy3p5] is an internal modification labeled on the nucleobase of deoxythymidine.
5BiotinTEG denotes Biotin-TEG attached to 5’-end
/3Cy3sp/ denotes Cy3 label attached to 3’-end
/3Cy5sp/ denotes Cy5 label attached to 3’-end
