# Supplementary material for: Tracking transcription–translation coupling in real time
Source: Nature. 2024 Dec 4;637(8045):487–95. doi: 10.1038/s41586-024-08308-w (PMC11711091; doi:10.1038/s41586-024-08308-w)
Supplement: Supplementary file 2 — Reporting Summary [file 41586_2024_8308_MOESM2_ESM.pdf]

nature portfolio

Corresponding author(s): Olivier Duss

Last updated by author(s): Oct 12, 2024

Reporting Summary

Nature Portfolio wishes to improve the reproducibility of the work that we publish. This form provides structure for consistency and transparency in reporting. For further information on Nature Portfolio policies, see our Editorial Policies and the Editorial Policy Checklist.

Statistics

For all statistical analyses, confirm that the following items are present in the figure legend, table legend, main text, or Methods section.

n/a	Confirmed	
☐	☒	The exact sample size (*n*) for each experimental group/condition, given as a discrete number and unit of measurement
☐	☒	A statement on whether measurements were taken from distinct samples or whether the same sample was measured repeatedly
☐	☒	The statistical test(s) used AND whether they are one- or two-sided<br>*Only common tests should be described solely by name; describe more complex techniques in the Methods section.*
☒	☐	A description of all covariates tested
☒	☐	A description of any assumptions or corrections, such as tests of normality and adjustment for multiple comparisons
☐	☒	A full description of the statistical parameters including central tendency (e.g. means) or other basic estimates (e.g. regression coefficient) AND variation (e.g. standard deviation) or associated estimates of uncertainty (e.g. confidence intervals)
☐	☒	For null hypothesis testing, the test statistic (e.g. *F*, *t*, *r*) with confidence intervals, effect sizes, degrees of freedom and *P* value noted<br>*Give P values as exact values whenever suitable.*
☒	☐	For Bayesian analysis, information on the choice of priors and Markov chain Monte Carlo settings
☒	☐	For hierarchical and complex designs, identification of the appropriate level for tests and full reporting of outcomes
☒	☐	Estimates of effect sizes (e.g. Cohen's *d*, Pearson's *r*), indicating how they were calculated

Our web collection on statistics for biologists contains articles on many of the points above.

Software and code

Policy information about availability of computer code

Data collection	Custom software implemented in Metamorph software package (Molecular Devices) by Cairn Research was used for single-molecule fluorescence microscopy data acquisition.
Data analysis	Single-molecule experimental data were analyzed using SPARTAN 3.7.0, tMAVEN and MATLAB R2021a and previous versions (MathWorks) using custom scripts which were previously made available publicly under an open source license at: https://github.com/puglisilab/Lapointe-2022-Nature. Figures were prepared in MATLAB, Adobe Illustrator, Excel 2016, ChimeraX 1.3 and Pymol (v. 2.4). Gel bands were integrated using ImageLab 6.1 software (BioRad).<br>Our Code availability statement reads: "The software used for single-molecule data processing and analysis is published72,73 and freely available online. Additional scripts for downstream processing were made available publicly previously under an open-source license74."

For manuscripts utilizing custom algorithms or software that are central to the research but not yet described in published literature, software must be made available to editors and reviewers. We strongly encourage code deposition in a community repository (e.g. GitHub). See the Nature Portfolio guidelines for submitting code & software for further information.

Data

Policy information about availability of data

All manuscripts must include a data availability statement. This statement should provide the following information, where applicable:

- Accession codes, unique identifiers, or web links for publicly available datasets
- A description of any restrictions on data availability
- For clinical datasets or third party data, please ensure that the statement adheres to our policy

Data availability. Structures were downloaded from the Protein Data Bank (https://www.rcsb.org/) using the accession codes shown in the main figures (6xdq, 6ztm and 6flq) or listed in the Source Data for Extended Data Fig. 3C,D. The published article includes all relevant data generated or analyzed during this study. The source data used in all the figures are presented in the Source Data file. All single-molecule traces, including transition assignments, are deposited in Zenodo with following link: https://zenodo.org/records/13271669. The raw data files are too large (several TBs of data) but are available upon request to O.D.

Research involving human participants, their data, or biological material

Policy information about studies with human participants or human data. See also policy information about sex, gender (identity/presentation), and sexual orientation and race, ethnicity and racism.

Reporting on sex and gender	N/A
Reporting on race, ethnicity, or other socially relevant groupings	N/A
Population characteristics	N/A
Recruitment	N/A
Ethics oversight	N/A

Note that full information on the approval of the study protocol must also be provided in the manuscript.

Field-specific reporting

Please select the one below that is the best fit for your research. If you are not sure, read the appropriate sections before making your selection.

☒ Life sciences ☐ Behavioural & social sciences ☐ Ecological, evolutionary & environmental sciences

For a reference copy of the document with all sections, see nature.com/documents/nr-reporting-summary-flat.pdf

Life sciences study design

All studies must disclose on these points even when the disclosure is negative.

Sample size	As customary in the field, we did not perform statistical analysis to predetermine sample size and no methods were used to predetermine sample size. As much single-molecule data as possible were collected to enable robust analyses. In principle, to obtain a rate constant from a single-molecule data set only a few molecules would be sufficient but the field usually reports 50-100 molecules per experiments. Therefore, we analyzed at least 100 events/molecules if possible and in most experiments several hundreds.
Data exclusions	Raw single-molecule time traces were excluded if they 1) consisted of more than one molecule, 2) lacked relevant fluorescence signals (for example, lack of Cy5, Cy3 or Cy3.5 signals corresponding to a fully assembled expressome), 3) were too noisy to unambiguously assign all the relevant events.
Replication	Quantitative measurements were reliably reproduced with 2-3 replicates (see extended Data Figures and Source Data) and were in agreement with data from other measurements, if possible.
Randomization	Samples were not randomized as this is not applicable to single-molecule biophysical and biochemical studies. Sample randomization is not applicable to biochemical experiments as for a specific experiment a single well-defined condition is measured. For data-evaluation the entire dataset was evaluated. For specific experiments, with much more molecules present that required for evaluation, a random selection of molecules was used for data-evaluation
Blinding	Investigators were not blinded as it does not involve clinical data, human or animal models or group allocation and is therefore not applicable to this study. The reasons that blinding is not applicable to biochemical experiments are that biochemical experiments rely on automated processes (such as a single-molecule microscopy measurement) and thus, are minimally affected by human bias. Furthermore, biochemical experiments are highly reproducible and can be verified by repetitions and measurements are very precise and thus have minimal observer bias.

Reporting for specific materials, systems and methods

We require information from authors about some types of materials, experimental systems and methods used in many studies. Here, indicate whether each material, system or method listed is relevant to your study. If you are not sure if a list item applies to your research, read the appropriate section before selecting a response.

Materials & experimental systems

n/a	Involved in the study
☒	☐ Antibodies
☒	☐ Eukaryotic cell lines
☒	☐ Palaeontology and archaeology
☒	☐ Animals and other organisms
☒	☐ Clinical data
☒	☐ Dual use research of concern
☒	☐ Plants

Methods

n/a	Involved in the study
☒	☐ ChIP-seq
☒	☐ Flow cytometry
☒	☐ MRI-based neuroimaging

Plants

Seed stocks

Report on the source of all seed stocks or other plant material used. If applicable, state the seed stock centre and catalogue number. If plant specimens were collected from the field, describe the collection location, date and sampling procedures.

Novel plant genotypes

Describe the methods by which all novel plant genotypes were produced. This includes those generated by transgenic approaches, gene editing, chemical/radiation-based mutagenesis and hybridization. For transgenic lines, describe the transformation method, the number of independent lines analyzed and the generation upon which experiments were performed. For gene-edited lines, describe the editor used, the endogenous sequence targeted for editing, the targeting guide RNA sequence (if applicable) and how the editor was applied.

Authentication

Describe any authentication procedures for each seed stock used or novel genotype generated. Describe any experiments used to assess the effect of a mutation and, where applicable, how potential secondary effects (e.g. second site T-DNA insertions, mosiacism, off-target gene editing) were examined.
